# Modified Long-Axis In-plane Technique for Radial Artery Cannulation in Children: A Randomized Controlled Trial

**DOI:** 10.3389/fmed.2021.780375

**Published:** 2022-02-07

**Authors:** Liu Yu, Heying Zhong, Yan Jiang, Wangping Zhang, Zhiwei Liu

**Affiliations:** ^1^Department of Anesthesiology, Women and Children's Hospital of Jiaxing University, Jiaxing, China; ^2^Department of Anesthesiology, Huadu District People's Hospital, Southern Medical University, Guangzhou, China; ^3^Department of Anesthesiology, Shanghai Children's Hospital, Shanghai Jiao Tong University, Shanghai, China; ^4^Department of Pediatrics, International Peace Maternity and Child Health Hospital, Shanghai Jiao Tong University School of Medicine, Shanghai, China

**Keywords:** radial artery, cannulation, ultrasound, children, success rate

## Abstract

**Background:**

Radial artery catheterization is a challenge for anesthetists in the pediatric population. The purpose of this study was to determine whether the modified long-axis in-plane (MLAX-IP) technique increased the success rate of radial artery catheterization in children.

**Methods:**

This study involved 80 children who required arterial catheterization and were randomly divided into the MLAX-IP group and dynamic needle tip positioning (DNTP) group (40 cases in each group). Radial artery catheterization was performed using either the MLAX-IP technique or the DNTP technique.

**Results:**

The first-attempt cannulation success rate was higher in the MLAX-IP group than in the DNTP group (95 vs. 80%, *P* = 0.043). The imaging time of the artery in the MLAX-IP group was longer than in the DNTP group (19.1 ± 3.1 vs. 9.6 ± 2.4 s, *P* < 0.001). While the total catheterization time was similar between the 2 groups (88.1 ± 23 vs. 86.9 ± 46.1 s, *P* = 0.475).

**Conclusion:**

The first-attempt cannulation success rate with the MLAX-IP technique is increased, while the total catheterization time is similar between the 2 groups and puncture-related complications are fewer.

## Introduction

Arterial cannulation was usually used for real-time hemodynamic monitoring in major surgery (1). Moreover, it is convenient to draw arterial blood for blood gas analysis. In the pediatric population, radial artery catheterization is a challenge for anesthetists due to the smaller diameter of the vessel (2, 3). Even for the skilled operators, it may cause vasospasm or hematoma at multiple attempts, thus significantly reducing the success rate of cannulation (4). At present, the common techniques of ultrasound (US)-guided vascular catheterization include short-axis, out of a plane (SAX-OOP), long-axis, in-plane (LAX-IP), and dynamic needle tip positioning (DNTP) techniques (5, 6). The first-attempt cannulation success rate was higher, cannulation time was longer and incidence of puncture-related complications was decreased using the LAX-IP approach compared with the SAX-OOP approach (7). DNTP technique can overcome the problem that it is difficult to accurately determine the location of the needle tip compared with the traditional SAX-OOP technique (8). A recent study has shown that the DNTP technique for radial artery catheterization is superior to the traditional SAX-OOP technique (9). DNTP technique makes the needle tip visible, while the LAX-IP technique makes the whole needle visible, it allows better visual control of the needle. It seems that the LAX-IP technique is more suitable for arterial cannulation because of its higher cannulation success rate, but its cannulation time is longer. As for arterial catheterization, it is still controversial which US technique (DNTP technique or LAX-IP technique) has more suitable for arterial catheterization. In this study, we applied the modified long-axis in-plane (MLAX-IP) technique to arterial cannulation. The purpose of this study was to explore whether the MLAX-IP technique increased the success rate of radial artery catheterization in children and did not extend the cannulation time.

## Materials and Methods

### Study Design

The study was conducted in accordance with the Declaration of Helsinki and was approved by the ethics committee of Shanghai children's hospital (approval No. 2019R043). Written informed consent was obtained from the children's parents or guardians. The trial was registered prior to patient enrollment in the Chinese Clinical Trials Registry (registration number: ChiCTR1900025254, principal investigator: JY, and date of registration: June 23, 2020). From August to December 2020, 80 children requiring invasive arterial blood pressure monitoring, ASA grade I-II, of either sex or age of 1–3 years, were enrolled in this study. Using a computer random number table, the children were randomly divided into the DNTP group and MLAX-IP group, 40 cases in each group.

In the operating room, electrocardiogram, non-invasive blood pressure, heart rate, and pulse oxygen saturation were monitored. Anesthesia was induced with sufentani l.3 μg/kg, propofol 3 mg/kg, and rocuronium 0.6 mg/kg, and was maintained with 2–3% sevoflurane. Radial artery catheterization was performed under US guidance in both groups. The children were placed in a supine position with the wrist extended for the best visualization of the artery. After sterilizing skin, a disposable protective sleeve on the US probe (Huasheng Medical Company, China, 15 L linear array probe, 5–13 MHz, depth 3 cm) was applied. The operator first chose the right radial artery for catheterization with a 24- or 22-G disposable arterial puncture needle (Bidi Medical Company, Jiangsu, China). After the sterile preparation, the timer started to count when beginning of US probe manipulation. If the radial artery catheterization failed within 10 min, the study was stopped. The operator could use other approaches for catheterization. All radial artery catheterization was performed by the same experienced anesthetists (performed more than 100 arterial cannulations).

#### DNTP Group

The procedure of the radial artery catheterization using the DNTP technique was as follows: First, the image of the radial artery was obtained via short-axis view, then the needle was inserted percutaneously at an angle of 30–40° until the needle tip appeared inside the artery lumen as a hyperechoic point. At that time the insertion of the needle was stopped, and the US probe was moved proximally along the radial artery until the needle tip disappeared from the short-axis view under US guidance. Then the needle was advanced for 3–5 mm until the needle tip reappeared inside the lumen of the artery under the US image. Repeating this process several times, the insertion angle was gradually decreased in order to keep the needle tip in the center of the cross-sectional lumen of the artery as much as possible. The needle tip was thus advanced for 3–5 mm. The needle core was then drawn out and the catheter connected to the pressure tubing and transducer for measuring blood pressure.

#### MLAX-IP Group

**Figure 1:**
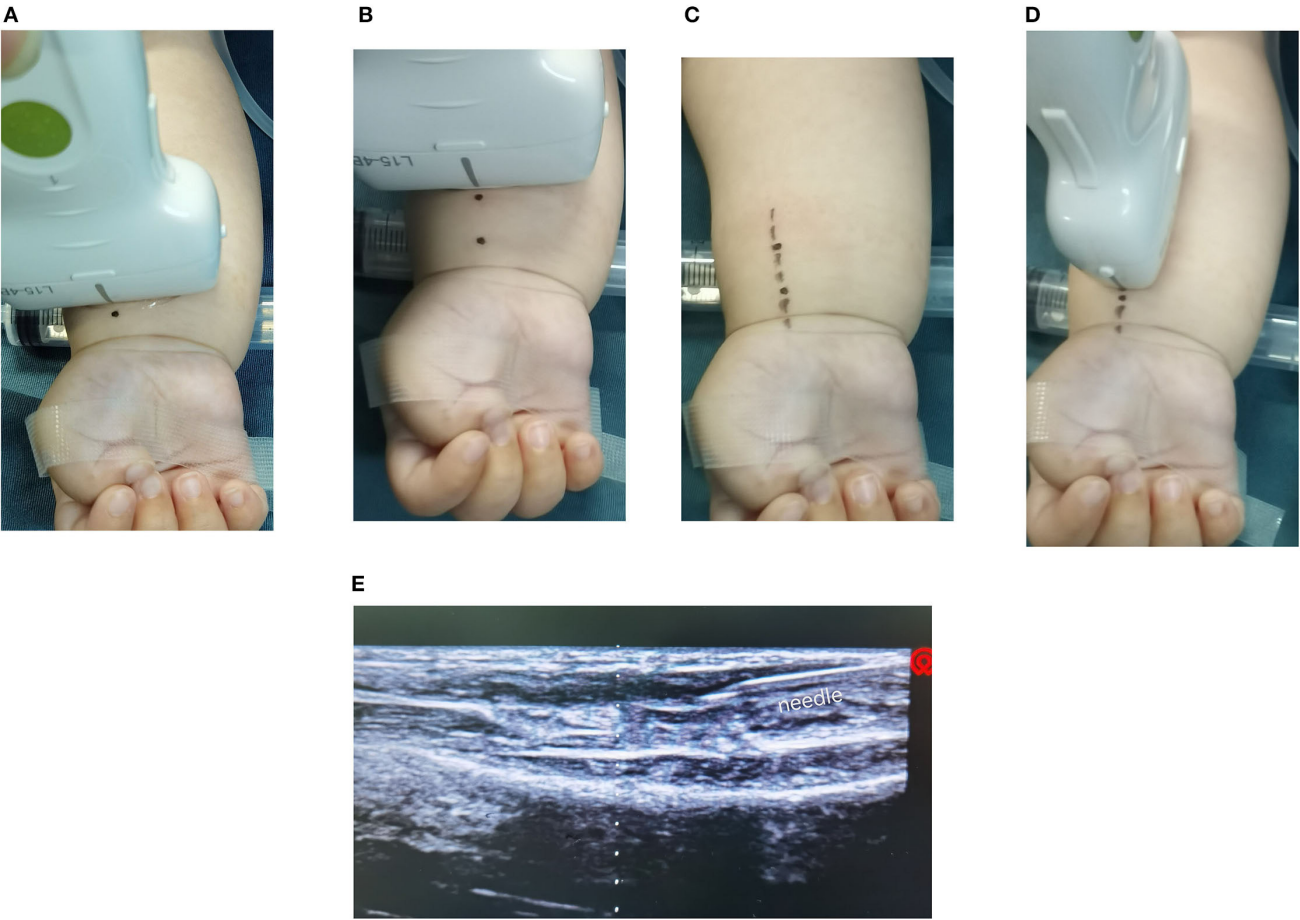
**(A–E)** The radial artery was marked on the skin using the MLAX-IP technique.

The radial artery marked on the skin using the short-axis technique is shown in (Figures 1A–E). In the supine position, the US probe was placed on the wrist. Using the short-axis view, the 2 middle points (1 cm of distance between the 2 points) of the radial artery were marked on the skin, then joined two points by a straight line. The middle mark of the US probe was placed in line with the straight line on the skin and an optimum long-axis view of the radial artery was obtained. The needle was then inserted percutaneously at an angle of 30–40° using the in-plane technique. The angle was readjusted using real-time imaging according to the relative position of the needle to the artery. As soon as a retrograde blood flow was obtained at the end of the needle, the needle was pushed forward for 3–5 mm inside the artery under real-time sonographic imaging. The catheter was eventually pushed forward into the lumen of the artery while the retrograde blood flow could still be noted at the end of the needle.

### Outcome Measures

The imaging time, total catheterization time, and first-attempt success rate of catheterization were recorded. The imaging time of the artery is defined as the time from the beginning of manipulating the US probe to the time at which the image of targeting radial artery was in the middle of the screen. The total catheterization time is defined as the time from the beginning of manipulating the US probe to the time when catheterization was successful, or the catheter was in the vessel lumen.

Puncture-related complications (the posterior wall puncture, infection, thrombosis, and puncture hematoma) were recorded. Hematoma is defined as the diffuse dark shadow around an artery under the US.

### Statistical Analysis

As the primary outcome was set as the first-attempt success rate of puncture. According to the previous study (8) and our pilot study, 36 samples were included in each group at α error of 0.05 and power of 0.8. The final sample size was increased to forty children to account for dropouts.

The data with normal distribution were expressed as mean ± *SD* and were tested by independent sample *t*-test. The data with non-normal distribution were expressed by inter-quartile range, and the significant difference was Wilcoxon Rank Sum Test. A *P* < 0.05 was defined as the significant difference.

## Results

### Children's Characteristics

**Figure 2:**
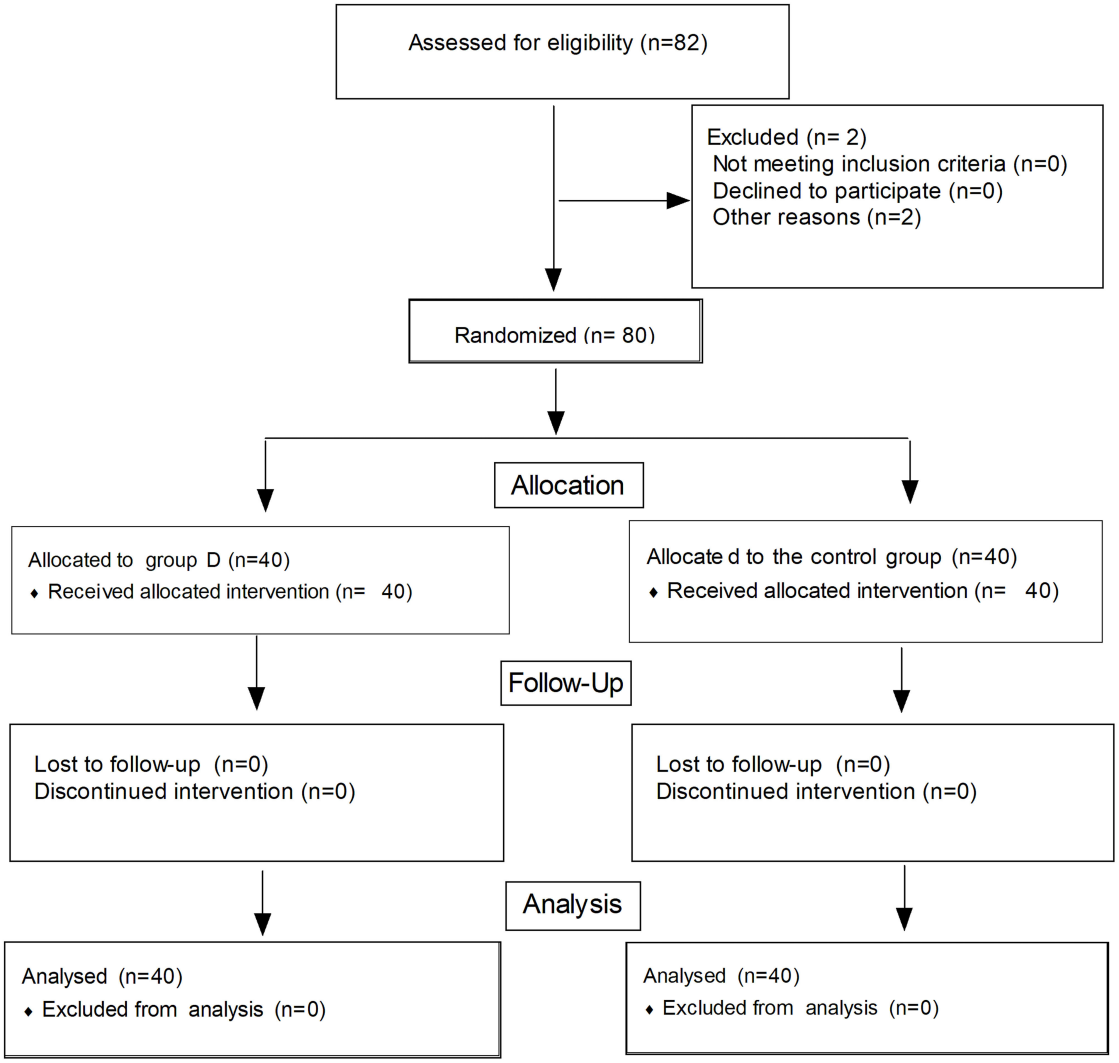
Flow diagram of the study.

Eighty-two children were enrolled in this trial and eighty children finished this study (Figure 2). There were no significant differences in age, gender, weight, the diameter of the artery, depth of skin to the artery wall, and mean arterial pressure between the two groups (*P* > 0.05, in Table 1).

**Table 1:** The characteristics of infants.

**Index**	**MLAX-IP group (*n* =40)**	**DNTP group** **(*n* = 40)**	***P-*value**
Sex (male/female)	21/19	25/15	0.366
Age (year)	2.7 ± 0.7	2.7 ± 0.9	0.796
Weight (kg)	13.1 ± 1.9	13.7 ± 2.2	0.159
Arterial diameter (mm)	2.25 ± 0.19	2.21 ± 0.18	0.371
Depth of artery (mm)	3.0 ± 0.6	3.0 ± 0.6	0.971
Mean arterial pressure (mmHg)	62.5 ± 4.2	63.4 ± 6.8	0.477

*Data are expressed as the mean ± standard deviation or number*.

### The Variables of Puncture

The variables of puncture are shown in Table 2. The first-attempt success rate of cannulation was higher in the MLAX-IP group than in the DNTP group (95 vs. 80%, *P* = 0.043). The imaging time of artery in the MLAX-IP group was longer than in the DNTP group (19.1 ± 3.1 vs. 9.6 ± 2.4 s, *P* < 0.001), while the total catheterization time was similar between the 2 groups (88.1 ± 23 vs. 86.9 ± 46.1 s, *P* = 0.475). There was a significant difference in posterior wall punctures between the 2 groups (2 vs. 8, *P* = 0.043), but the incidence of the other puncture-related complications was similar (*P* > 0.05).

**Table 2:** Comparison of the variables of puncture between the two groups.

**Index**	**MLAX-IP group (*n* = 40)**	**DNTP group** **(*n* = 40)**	***P-*value**
Image time of artery (*s*)	19.1 ± 3.1	9.6 ± 2.4	0.000
Total catheterization time (*s*)	81.1 ± 23.0	86.9 ± 46.1	0.475
First-attempt success rate (%)	38 (95%)	32 (80%)	0.043
Posterior wall punctures (*n*)	2 (5%)	8 (20%)	0.043
Hematoma (*n*)	1 (2.5%)	4 (10%)	0.359
Infection (*n*)	0	0	1
Thrombosis (*n*)	0	0	1

*Data are expressed as the mean ± standard deviation or number*.

## Discussion

Radial artery catheterization is a challenge to anesthetists in children because of the small vessel size. In this study, we found that the first-attempt success rate of cannulation with the MLAX-IP technique was higher in children compared with the DNTP technique, but the total catheterization time was similar between the 2 groups and puncture-related complications were fewer.

In this study, the first-attempt success rate of radial artery catheterization was higher using MLAX-IP compared with the DNTP technique. The literature showed that the first-attempt success rate of radial artery cannulation with the DNTP technique was significantly higher than that with the LAX-IP technique (10). DNTP technique made the needle tip visible, which facilitated inserting the needle into the center of the artery thus reducing the risk of failure when pushing the arterial catheter (11). In the present study, the middle mark of the long-axis of the US probe was placed to parallel to the straight line on the skin when using the MLAX-IP technique, and the whole needle was visible so that the needle would penetrate straight away into the center of the artery lumen increasing thus the cannulation success rate. If the needle tip was not located in the center of the artery lumen, the cannulation success rate would be decreased. In this study, the methods used for searching the long-axis view were different from the other methods. The common methods of searching the long-axis view are as follows: firstly, search image of the artery under the short-axis view, then rotate the US probe at 90 degrees. In this study, we needed to mark the middle points of the radial artery at two different positions with a distance of 1 cm on the skin under short-axis view, and joined the two points by a straight line, then placed the middle mark of the US probe parallel to the straight line with LAX-IP technique. Therefore, the imaging time of the artery in the MLAX-IP group was longer than that in the DNTP group (*P* < 0.05). However, there were no significant differences in the total catheterization time between the DNTP group and the MLAX-IP group. Although the MLAX-IP technique took more time on the imaging time of the artery, the total cannulation time was similar between the 2 groups. As the whole needle was visual in the long-axis plane view, the needle was quickly inserted into the targeting artery. Quan et al. (12) compared a modified SAX approach with a conventional LAX approach and found that the cannulation success rate on the first attempt was significantly higher when using the modified SAX-OOP technique. Their results were contrary to our findings. In Abdelbaser's study, the first-attempt success rate of femoral artery catheterization with the LAX-IP approaches was higher (*P* = 0.048) in neonates and infants compared to the SAX-OOP approaches, the total catheterization time was significantly shorter using the LAX-IP approaches, and the rate of complication was similar between the two approaches (13). In our study, the first-attempt success rate of cannulation by the MLAX-IP approach was higher than by the DNTP approach (*P* = 0.043). LAX-IP approach allowed better visual control of the needle throughout its whole length and the relative position of the needle to the posterior wall of the artery (14). Therefore, the MLAX-IP technique was more suitable for radial artery cannulation than the DNTP technique in terms of the cannulation success rate and posterior wall puncture.

The whole needle is not visualized by the traditional SAX-OOP technique, which may lead to the posterior wall punctures of the artery and hematoma, even the failure of cannulation (9, 15). Accidental injury of posterior wall of artery did not be completely avoided when DNTP technique was used. The whole needle was visualized with the MLAX-IP technique, which avoided the posterior wall punctures of the artery as far as possible. The success rate of cannulation was related to the position and direction of the needle to the artery. The success rate of catheterization would be increased if a needle tip was inserted into the center of the artery lumen.

### Limitations

First, the operator should master DNTP and LAX-IP techniques under the US, and the personal skills and experience of operators may affect the results. Due to the small sample, puncture-related complications require further study on large samples.

## Conclusion

This study indicates that the MLAX-IP technique may increase the first-attempt success rate of cannulation, reduce the incidence of posterior wall punctures, and not increase total catheterization time in children, compared with the DNTP technique.

## Data Availability Statement

The raw data supporting the conclusions of this article will be made available by the authors, without undue reservation.

## Ethics Statement

The study was conducted in accordance with the Declaration of Helsinki and was approved by the Ethics Committee of Shanghai children's hospital (approval No. 2019R043). The patients/participants provided their written informed consent to participate in this study.

## Author Contributions

LY, WZ, ZL, and YJ: these authors designed the study. HZ and LY: these authors analyzed the data for the manuscript. All authors contributed to the article and approved the submitted version.

## Conflict of Interest

The authors declare that the research was conducted in the absence of any commercial or financial relationships that could be construed as a potential conflict of interest.

## Publisher's Note

All claims expressed in this article are solely those of the authors and do not necessarily represent those of their affiliated organizations, or those of the publisher, the editors and the reviewers. Any product that may be evaluated in this article, or claim that may be made by its manufacturer, is not guaranteed or endorsed by the publisher.
